# Cell-free DNA as a biomarker in cancer

**DOI:** 10.20517/evcna.2022.20

**Published:** 2022-08-02

**Authors:** Robert H. Eibl, Markus Schneemann

**Affiliations:** ^1^c/o M. Schneemann, Department of Internal Medicine, Hospitals of Schaffhausen, 8208 Schaffhausen, Switzerland.; ^2^Department of Internal Medicine, Hospitals of Schaffhausen, 8208 Schaffhausen, Switzerland.

**Keywords:** Cell-free DNA, cfDNA, circulating tumor DNA, ctDNA, biomarker, liquid biopsy, MRD, cancer

## Abstract

Translational research of liquid biopsy is just at the edge of routine clinical application: an emerging validity of circulating tumor DNA (ctDNA) tests suggests its use for earlier cancer detection and better monitoring of minimal residual disease (MRD) and resistance development, thus offering earlier guidance for therapy choices with the intent to cure cancer. In this review, we focus on ctDNA as an advanced and standardized validated marker in liquid biopsy. We also discuss what will be needed to reach the new milestone of personalized (precision) medicine to be used as a common standard of care. We summarize recent developments of cell-free DNA (cfDNA) and its clinical use as a biomarker in cancer.

## INTRODUCTION

**Figure 1:**
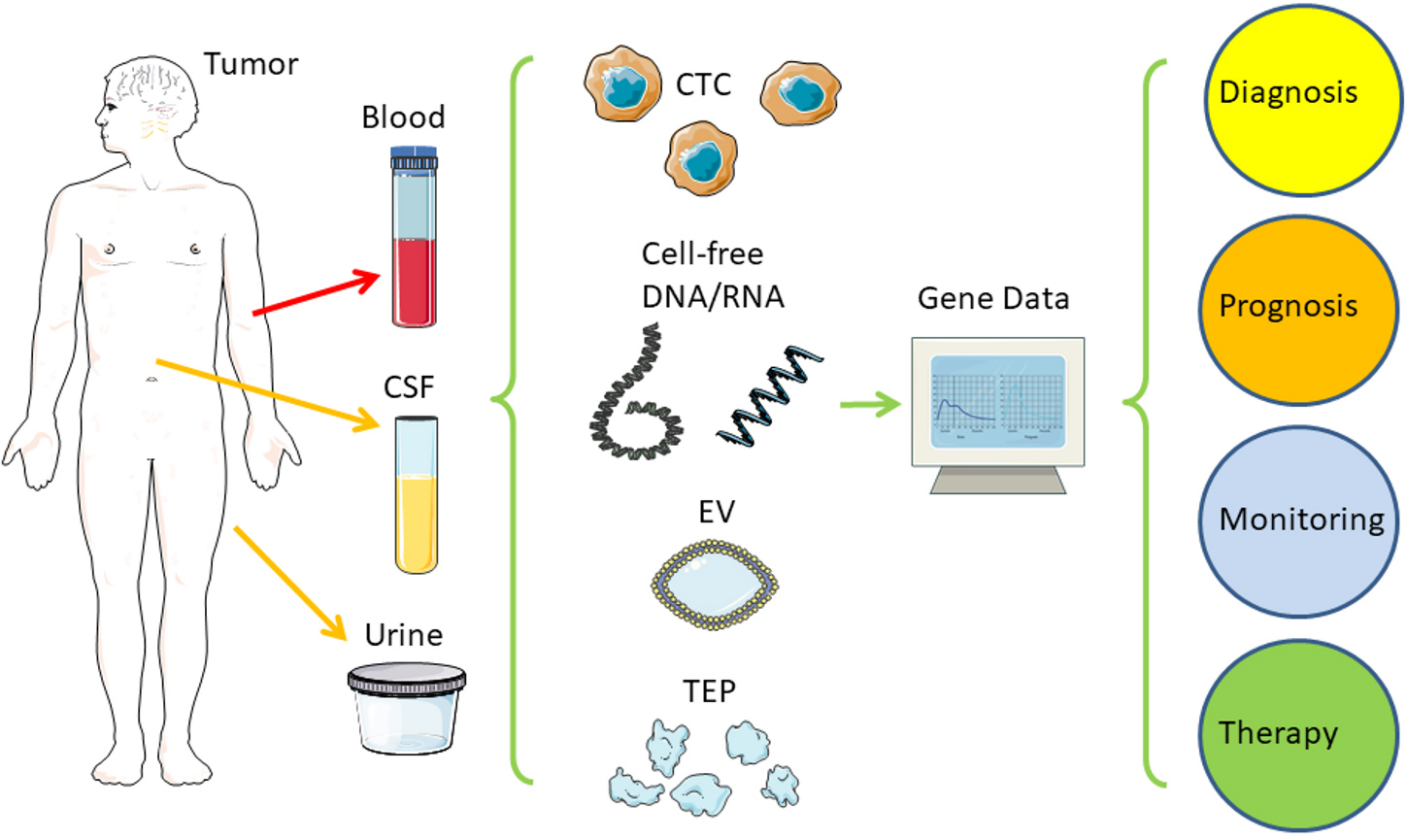
Liquid biopsy. Distant from the original tumor, samples from blood, CSF, or urine can serve as an easily acquired and low-risk source of tumor-derived nucleic acids (RNA and DNA) for further analysis to predict and monitor tumor progression and treatment response. CSF: Cerebrospinal fluid; CTC: circulating tumor cell; EV: extracellular vesicle; TEP: tumor educated platelets. Created/modified with SMART^[1]^, licensed under Creative Commons Attribution 3.0 Unported License^[2]^.

**Figure 2:**
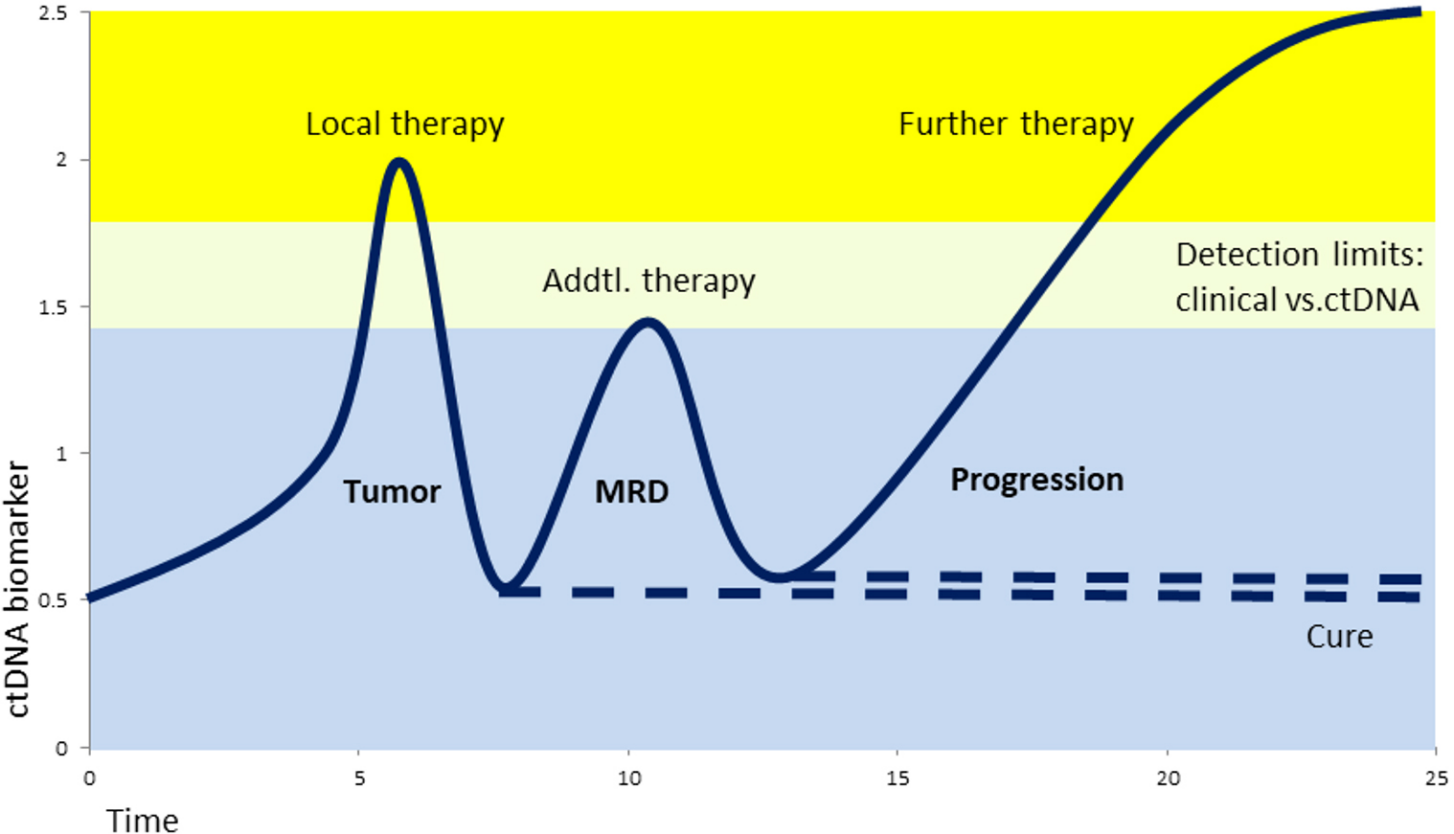
Hypothetical ctDNA biomarker levels during tumor development and therapy. After tumor removal, the biomarker level drops significantly and will remain low in the case the patient is cured. Early detection of minimal residual disease (MRD) by ctDNA allows additional treatment (e.g., chemo-, radio-, or hormone therapy) with the intent of cure, much earlier than with clinical imaging methods.

Key elements of clinical routine with tumor patients include cancer detection and diagnosis as well as monitoring tumor development and treatment response. Usually, an interdisciplinary approach is necessary with complementing areas, such as medical imaging, tissue biopsy, histopathology, surgery, chemotherapy, and radiotherapy. Over decades, measurements of protein tumor markers in the blood evolved to the current standard, but translational research offers improvement: circulating cell-free DNA (cfDNA) allows easy and early access to information on tumor evolution and treatment response at low risk of injury, infection, or wound healing, compared to repeated tissue biopsies or delayed medical imaging, thus enabling improved therapy decisions and avoidance of over-treatment which improves patients’ quality of life [Figures 1 and 2]. Circulating tumor cells (CTCs) were first described in an autopsy over 150 years ago by Ashworth, who found tumor cells in the blood microscopically identical to the tumor cells in metastatic skin lesions [Table 1]^[3]^. When in 1889, over 130 years ago, the surgeon Stephen Paget presented his “seed and soil” theory, it was already known that tumor cells were able to disseminate via blood circulation. Metastatic cancer cells (“seeds”) can leave their site of origin and enter the bloodstream to grow as secondary tumors in favorable environments (“soil”) quite distant from the primary tumor^[4]^. With his model, Paget explained a 15-fold higher frequency of breast cancer metastasis to the liver compared to the spleen. Although only a very small fraction of tumor cells within the blood gives rise to secondary tumors, it is well accepted nowadays that circulating tumor cells (CTCs) indicate tumor progression and an increased risk of metastasis [Figure 1]^[6]^. Over the last two decades, the detection and analysis of these CTCs have improved considerably enough to be tested in clinical settings [Table 1]^[28]^. In 2004, Allard and colleagues were able to detect and count CTCs in blood from many patients with prostate, breast, ovarian, colorectal, lung, and other cancers^[11]^. In the same year, Cristofanilli and colleagues showed that an elevated number of CTCs before treatment of metastatic breast cancer can serve as an independent prognostic marker for a worse outcome, namely a shortened duration of both progression-free survival (PFS) and overall survival (OS)^[12]^. Molecular profiling of DNA extracted from CTCs from non-small cell lung cancer (NSCLC) patients during treatment allowed monitoring of tumor evolution by mutations in the epidermal growth factor receptor (EGFR) gene related or unrelated to therapy resistance^[14]^. As an alternative to the technical obstacles and the low sensitivity of fishing for the extremely rare CTCs, the cell-free fraction of blood can also be used to extract DNA, which then also allows the finding of tumor-specific mutations. In 1948, French scientists reported free nucleic acids in blood, but they did not relate them to any disease^[5]^. In 1977, Leon and colleagues used polyclonal antiserum from patients with systemic lupus erythematosus (SLE), an autoimmune disease generating antibodies against DNA, to detect increased values of free DNA in about half of their cancer patients^[7]^. Normal values for the other half of the patients were explained by the selection of patients before radiotherapy, but who already had their tumors surgically removed and/or had chemotherapy, resulting in an already reduced tumor mass. Despite significantly higher levels in patients with metastatic disease, no correlation was found regarding the size or location of the tumors. However, radiation therapy was able to decrease the levels of cfDNA significantly for lymphoma, lung, ovary, uterus, and cervical tumors, as well as to a lesser extent, glioma, breast, colon, and rectal tumors. Decreases in cfDNA levels went along with improved clinical conditions, such as lower tumor size and less pain, whereas increasing or unchanged levels correlated with worse conditions and a lack of response to treatment. The amount of ctDNA has been shown to markedly decrease after surgery or chemotherapy, suggesting the usefulness of ctDNA as a tumor marker [Figure 2 and Table 2]^[15]^. The term “liquid biopsy” was originally coined in 2010 by Pantel and Alix-Panabières for several methods of CTC detection and analysis, but it was also used for ctDNA or any other analysis of tumor-derived material from biofluids, e.g. blood, cerebrospinal fluid (CSF), or urine^[18,30]^. Recently, these methods of tracking cancer in liquid biopsy are considered one of the milestones in cancer research of the last two decades^[28]^.

**Table 1:** Historical timeframe and major developments of liquid biopsy

**Year**	**Author**	**Probe **	**Method**	**Tumor**	**Milestone**
1868	Ashworth^[3]^	CTC	Microscopy, case report	Skin metastasis of unknown primary, “liquid autopsy”	First report on tumor cells in blood; post mortem; microscopically identical cells in metastatic lesions
1889	Paget^[4]^	CTC	Autopsy	Breast cancer, postulated	“Seed and soil” theory of cancer metastasis
1948	Mandel and Métais^[5]^	cfDNA	Blood analysis	Not related to cancer, healthy blood donors	First report of (cell-free) nucleic acids in blood
1975	Fidler^[6]^	CTC	Experimental metastasis assay	B16 melanoma cell lines	Only a small fraction of intravenously injected tumor cells give rise to metastasis in mouse models
1977	Leon *et al*.^[7]^	cfDNA	Radioimmunoassay for free DNA in serum	Various cancers	First report on increased cfDNA levels in some cancer patients; correlation with therapy response
1989	Lo *et al*.^[8]^	cfDNA	PCR of Y chromosome-specific sequence; cfDNA/blood	Not related to cancer	Sex determination of fetus in pregnant women
2001	Reya *et al*.^[9]^	CTC	Applying hematopoietic stem cell knowledge to heterogeneity of cancer cells	Solid tumors and leukemia, migratory CSC	Cancer stem cell theory
2003	Balaña *et al*.^[10]^	ctDNA	Methylation-specific PCR of MGMT, p16, DAPK, RASSF1A	GBM	Detection of methylated MGMT in serum highly predictive for response to BCNU chemotherapy
2004	Allard *et al*.^[11]^	CTC	CellSearch™	Prostate, breast, ovarian, CCR, lung, and other cancers	Detection of CTCs in 7.5 mL of blood samples
2004	Cristofanilli *et al*.^[12]^	CTC	CellSearch™ Amount of CTC	Metastatic breast cancer	Independent predictive marker: reduced PFS and reduced OS
2005	Diehl *et al*.^[13]^	ctDNA	dPCR, BEAMing	Advanced CRC	APC mutations in plasma
2008	Maheswaran *et al*.^[14]^	CTC	Molecular profiling EGFR mutations	NSCL	Monitoring therapy
2008	Diehl *et al*. ^[15]^	ctDNA	Mutations	Colorectal cancer	Amount and presence/absence as an independent tumor marker, monitoring
2008	Cohen *et al*.^[16]^	CTC	CellSearch Clinical study	Colorectal cancer	Enumerating CTC
2008	De Bono *et al*.^[17]^	CTC	Clinical study	Prostate Cancer	Enumerating, the first demonstration that CTCs are the most accurate and independent predictor of OS in metastatic prostate cancer
2010	Pantel and Alix-Panabières^[18]^	CTC	Concept of analyzing tumor cells in body fluids	All cancers	Coined the term “liquid biopsy”
2010	Calverly *et al*.^[19]^	TEP	Platelet mRNA	Metastatic NSCL	Downregulation of gene expression in platelets
2013	Dawson *et al*.^[20]^	CTC + ctDNA (comparison)	Side-by-side disease monitoring	Metastatic breast cancer undergoing treatment	Sensitivity: ctDNA>CTC ctDNA level correlates with treatment response
2014	Bettegowda *et al*.^[21]^	ctDNA	Digital PCR, sequencing	14 tumor types	ctDNA detectable for most tumors outside the brain
2014	Sullivan *et al*.^[22]^	CTC	“Negative depletion” CTC-iChip (removing leukocytes from blood)	GBM (usually not metastatic)	Surprising and frequent detection of CTCs in brain tumors
2015	Mazel *et al*.^[23]^	CTC	CellSearch	Breast cancer	PD-L1 detection for immune checkpoint inhibition
2016	Tie *et al*.^[24]^	ctDNA	Sequencing	Colon cancer stage II	Detection of MRD after surgery; prediction of recurrence after chemotherapy
2016	Donaldson and Park^[25]^	ctDNA	Clinical studies	NSCLC	First FDA^[29]^ and EMA approval to use ctDNA for EGFR-targeted therapy
2018	Cohen *et al*.^[26] ^	ctDNA, plus proteins from blood	CancerSEEK, detecting mutations in 1933 loci of 16 genes; combined with protein tumor markers	8 cancer types	Blood screening test for several common cancers
2020	Lennon *et al*.^[27]^	ctDNA, protein markers plus PET-CT	Prospective 16 gene locations, 8 tumor proteins, PET-CT	Multi-cancer screening of 10,000 women with no known cancer	Multi-cancer blood testing combined with PET-CT

CRC: Colorectal cancer; CSC: cancer stem cell; CTC: circulating tumor cell; ctDNA: circulating tumor DNA; EGFR: epidermal growth factor receptor; EMA: European Medicines Agency; enumerating: counting; FDA: US Food and Drug Administration; PET-CT: positron emission tomography-computed tomography; TEP: tumor educated platelets.

**Table 2:** Principles of liquid biopsy (ctDNA or CTC) in clinical settings

**Liquid Biopsy**	**Pro**	**Con**	**Clinical utility**
CTC	Sufficient sensitivity for some advanced-stage cancers, incl. metastases	Sensitivity limited: - Early-stage cancers - Cancer screening - Many advanced-stage cancers	Prognosis in many metastatic settings: - Breast - Prostate - Colorectal carcinoma
	Validated for specific applications (enumeration)	Not fully standardized, difficult methods / for many applications; rare CTCs; very few, centralized high-tech laboratories needed	Prediction of relapse after treatment
	High specificity (mutations)	Sophisticated technology, no easy and common standards	Live CTCs useful for drug screening and functional assays
ctDNA	Standardized, common methods: - blood drawing/handling (routine in hospitals AND practice) - DNA extraction (decentralized laboratories) PCR/sequencing/analysis	Sensitivity too low: - Early-stage cancers - Cancer screening - Some advanced-stage cancers	- Diagnosis - Prognosis - Monitoring: Tumor evolution Relapse Therapy resistance (e.g., EGFR mutations) (all complementing tissue biopsy, medical imaging)
	High specificity (often close to 100% with tumor-related mutations)	Limited biological and clinical relevance of many detected mutations (not every mutation is relevant or druggable)	Personalized, precision medicine: early detection of druggable mutations
	Fast/real-time monitoring of relapse or resistance after therapy		
	Medium sensitivity for advanced cancer		

Over the last two decades, liquid biopsy has included numerous methods to search for cancer-derived markers by analyzing biofluids, e.g. blood, CSF, or urine, i.e. samples taken quite distant from both the original tumor and potential metastases, generally with much easier and less risky access [Figure 1]^[31]^. This led to a new definition of liquid biopsy with different circulating biomarkers in different body fluids^[30]^. Basically, liquid biopsy targets circulating tumor cells (CTC), or circulating tumor DNA (ctDNA), which is part of the whole cfDNA [Table 2]. Other examples include the analysis of extracellular vesicles (EVs) and RNA, such as microRNA (miRNA, miR) or, very recently, circular RNA (circRNA)^[32]^. The common goals are the use in screening, i.e. early detection and diagnosis of cancer with the chance for earlier and more curative treatments, as well as the use in monitoring after therapy and a better quality of life. The vision is a quick, reliable, and easy-to-repeat test for all patients to track tumor progression in real-time, as well as monitor the response to treatment to allow earlier detection of resistance, relapse, and metastasis and adapt clinical decisions accordingly. In addition, the presence of tumor markers after initially successful therapy should confirm minimal residual disease (MRD) in the absence of other markers and lead to additional treatments before the tumor becomes incurable^[33]^. The current standard for most solid cancers consists of a more invasive tissue biopsy and/or surgical removal of all or most parts of the tumor with a thorough microscopic analysis by a tumor pathologist. Genetic molecular profiling of the primary tumor tissue may be included. In 2016, a new WHO classification of tumors of the nervous system introduced molecular and genetic profiling as standard practice for the analysis of some brain tumors such as medulloblastomas and glioblastomas^[34]^. Depending on the tumor location and the multi-morbidity of a patient, obtaining tumor tissue repeatedly can be difficult and unsuitable for long-term monitoring of tumor development. Furthermore, transthoracic lung biopsies can lead to, although rare, iatrogenic dissemination of cancer cells^[35]^, and fine-needle biopsies have been reported to increase CTCs in prostate cancer^[36]^. The snapshot of a tissue biopsy from a primary tumor may also miss minor, but progressing, tumor clones; it may also not represent metastatic clones with perhaps different molecular profiles for treatment resistances^[37]^. Therefore, liquid biopsy carries the potential to be the next gold standard for monitoring cancer progression. Following various research approaches to the detection and cultivation of CTCs, also leading to a better understanding of tumor progression and metastasis, hundreds of emerging clinical studies are continuing to develop new standards for monitoring melanoma, breast, prostate, colon, and head and neck cancers^[38-46]^. In this review, we focus on another part of liquid biopsy, cfDNA, which is reliably much easier to access and does not need elaborate methodologies to capture the extremely rare CTCs.

## LIQUID BIOPSY MARKERS

ctDNA and CTCs are currently more established than EVs and tumor-educated platelets (TEP) as tools in liquid biopsy [Figures 1 and 2 and Table 1]. In this review, our main focus is on ctDNA and its clinical application and potential. For completion and comparison, we mention CTCs, EVs, and TEPs only briefly.

### Cell-free DNA and circulating tumor DNA

The total amount of non-cellular DNA in samples such as blood is called cfDNA, which for tumor patients contains all tumor-derived DNA in various amounts, i.e. ctDNA.

cfDNA in blood is highly fragmented with a peak of 166 bp length and many smaller peaks of 10 bp less or more in healthy individuals. Interestingly, the fragment size of the tumor-derived ctDNA appears to be shifted to a lower peak at 146 bp, which allows for selection strategies to increase the relative amount of tumor-derived ctDNA and to increase the sensitivity. Most of the cfDNA is considered to come from leukocytes, such as granulocytes, lymphocytes, and monocytes, and to a lesser extend from other cells (muscle cells, endothelial cells, epithelial cells, fibroblasts, mesenchymal stem cells, and hepatocytes)^[47]^; interestingly, physical activity can trigger cfDNA release almost exclusively from granulocytes (up to 2-20-fold increase) and may affect liquid biopsy^[47]^. Tumor-derived ctDNA can come from apoptotic or necrotic parts of the primary tumor, as well as from metastases and CTCs. ctDNA often represents only an extremely small fraction of the total cfDNA, especially at early stages and after successful therapies. Therefore, detection of low concentrations of ctDNA remains the major challenge for widespread tumor screening and treatment monitoring. Several key technologies have been developed and applied to detect potential micro-metastases and local relapse before clinical and radiological manifestation. This includes several ways of targeted sequencing of known mutations as well as non-targeted, i.e., genome-wide analysis, and fragmentomics^[48]^.

#### Targeted analysis

Targeted analysis aims to identify tumor-specific mutations or methylations in ctDNA. Techniques include digital PCR (dPCR), BEAMing (beads, emulsions, amplification, magnetics), safe-sequencing system (Safe-SeqS), cancer personalized profiling by deep sequencing (CAPP-Seq), and tagged-amplicon deep sequencing (Tam-Seq). ctDNA can also be analyzed for its relative amount from total cfDNA to serve as a surrogate for tumor burden. This allows risk assessment and staging, as well as early monitoring of therapy response or treatment failure. Tight monitoring can detect mutations as targets or escape mechanisms and supports clinical decision making based on tumor evolution and resistance development. Specific mutations in the EGFR gene in ctDNA allow treatment of NSCLC with a tyrosine kinase inhibitor (TKI)^[29,49]^. Activating mutations of phosphatidylinositol-3-kinase catalytic subunit alpha (PIK3CA) are used to guide treatment in breast cancer. Resistant tumor cells can escape from therapies by gaining new mutations and clonal selection of the fittest tumor cells. For example, KRAS mutations can develop as a resistance mechanism during EGFR-targeted therapy for patients with CRC^[50,51]^.

DNA methylation is an epigenetic mechanism to regulate gene expression by adding methyl groups to the DNA^[52]^. In contrast to normal tissue, tumors can show a reversed hyper- and hypomethylation pattern, which can be important for tumor development and progression, as well as for clinical management of patients. Since 2003, typical methylation patterns of tumor DNA have been used to identify ctDNA in blood samples, e.g. MGMT relevant for brain tumors as an actionable target^[10,53,54]^. More recently, hydroxymethylation profiling with detection of 5-hydroxymethylcytosine (5hmC) as a less known molecular marker of epigenetics was applied in liquid biopsy of lung, pancreatic, and hepatocellular cancer (HCC)^[55]^. Lung cancer was characterized by a loss of 5hmC, whereas HCC and pancreatic cancer showed disease-specific changes, thus providing information about tumor type and stage.

#### Non-targeted analysis

Without prior information on specific mutations, non-targeted approaches try to investigate the entire genome, e.g., with whole exome sequencing (WES), whole genome sequencing (WGS), detection of copy number aberrations (CNA), and others^[56]^. This also allows the detection of subclones evolving under treatment or during natural tumor progression. Unfortunately, low amounts of ctDNA from patients without relapse or metastasis affect sensitivity and utility. Some of these techniques can detect point mutations not previously found in the primary tumor, but with specific value options for treatment and prognosis^[57]^.

#### Fragmentomics

The size of tumor-derived cfDNA from plasma tends to be shorter than normal background cfDNA^[58]^. These differences in fragmentation of DNA have been associated with reduced levels of a secreted DNASE1-like nuclease, DNASE1L3, in many tumor types (breast, colorectal, lung, gastric, head and neck non-squamous cell, and liver cancers)^[59,60]^. Future studies will have to link and translate the biology of circulating DNA and nucleases to include fragment size, end motifs, and jagged (single-stranded) ends into clinical applications in tumor biology^[48]^.

### Other types of liquid biopsy: CTCs, EVs, TEPs

Over the last two decades, CTCs entered clinical applications to detect and count them, as well as to determine their genetic profile for prognostics and clinical decision making [Figure 1 and Table 1]. CTCs are usually rare, but they often correlate and contribute to metastatic progression, although only a fraction of CTCs gives rise to metastasis^[6]^. Despite major advantages of CTCs in diagnostics, prognostics, monitoring, and as guides to therapy choice and delivery schedule, widespread use appears to be still further away. Currently, only a few highly equipped labs supported by major research funds seem to be able to collect and analyze CTCs. A common standard is not established. With CellSearch, the Food and Drug Administration (FDA) and the European Medicines Agency (EMA) approved the first liquid biopsy in 2004^[29]^. Originally, CellSearch was approved as a diagnostic tool to detect and count CTCs in blood samples to predict outcome (PFS and OS) only in metastatic breast cancer, but it later was expanded to monitor metastatic breast, colorectal, and prostate cancer patients.

miRNA are noncoding, 20-24 nucleotides long RNA molecules derived from just 1% of the whole genome. They are involved in the regulation of stability and translation of mRNA in health and disease. The potential effects of up to 1900 miRNAs are not fully understood. They can be found up- or downregulated in serum, EVs, and CTCs, as well as in urine^[61]^. Future studies may also include further RNA molecules, such as larger and more stable circular RNA (circRNA). circRNA can serve as an antagonistic sponge for miRNAs and can be involved in gene regulation of tumor cells. However, common standards are needed to be validated, especially since dysregulation of miRNA and circRNA may also be found in inflammatory diseases.

Tumors and normal cells can release small EVs, which contain typical proteins, DNA, and RNA. The intact cell membrane protects the enclosed compounds against degrading enzymes, such as RNAses, from outside the vesicle. Therefore, EVs can be analyzed for the potential markers. Isolation of EVs from blood is the method of choice for most solid tumors. As an exception, CSF to analyze EVs from brain tumors can give better results compared to blood samples for tumors growing close to the ventricles and due to a better signal-to-noise ratio for tumor *vs.* non-tumor EVs^[62]^.

Blood platelets are derived as anucleated cytoplasmic fragments from megakaryocytes. They can react to activation of membrane receptors and outside-in signaling and have been shown to facilitate metastasis via several mechanisms, including protecting tumor cells within the circulation from immune cells and shear stress, supporting the adhesion to endothelium through adhesion receptors, and releasing of angiogenic and mitogenic growth factors at sites of metastasis. Surprisingly, tumors can alter the RNA profile of platelets, leading to the term tumor educated platelet (TEP)^[63]^. The mechanism remains to be elucidated. TEPs from lung, brain, and breast cancer patients have been shown to be distinct from those with inflammatory or other diseases^[19,64]^.

## CLINICAL STUDIES

Currently, the registry ClinicalTrials.gov from the NIH^[65]^ finds over 900 clinical trials related to the search term “ctDNA” (911 as of 15 March 2022), most of them are ongoing (with 648 “not yet recruiting”, “recruiting”, “enrolling by invitation”, or “active, not recruiting”) and only a minority (95) listed as “completed”, the remaining others being declared as “terminated”, “suspended”, or “unknown status”. Many of these studies are not originally intended to prove the use of ctDNA as a possible measurement of treatment response for new therapy schemes, but they just routinely integrate the ctDNA analysis as an early detection arm for success or failure of the treatment, i.e., as a complement, or when data from tissue biopsy are missing. However, some of the studies can use the potential of early detection of MRD, often months before the clinical manifestation of local relapse or metastasis [Table 3]; this lead time allows an earlier adaptation of the therapy decisions, sometimes with the intent to cure. It indicates the emerging role of ctDNA measurements in clinical settings, which may become the new gold standard, especially when such treatment studies will become new therapy regimes - and then may need or accept ctDNA as necessary control.

**Table 3:** Examples of ctDNA studies for screening or monitoring different cancers and stages, as well as treatment response

**Year**	**Author**	**Tumor**	**Method**	**Findings**
2008	Diehl *et al*.^[15]^	CRC	Quantification of ctDNA by patient-specific mutations	Prediction/exclusion of relapse after surgery for at least a year (no long-term follow-up)
2015	Garcia-Murillas *et al*.^[66]^	Early-stage breast cancer	Monitoring of patient-specific mutations by dPCR from cfDNA	Monitor for MRD, prediction of metastatic relapse after initial therapy (pilot study)
2015	Olsson *et al*.^[67]^	Breast cancer	Whole genome sequencing of primary tumor, rearrangements by dPCR in plasma	ctDNA quantity predictive of poor survival
2016	Donaldson and Park^[25,29]^	Advanced NSCL	cobas EGFR Mutation Test v2	First ctDNA-based treatment selection EGFR mutation, exons 18-21 (for Erlotinib)
2017	Phallen^[68]^	CRC, breast, lung, ovarian, cancer	TEC-seq of cfDNA	Detection of early stage tumors
2017	Abbosh *et al*.^[82] ^	Early-stage NSCLC, TRACERx	ctDNA gene profiling with multiplex-PCR NGS	Identify post-operative relapse
2017	Ng *et al*.^[70]^	CRC	PPS-ctDNA, multiplex-PCR	Monitoring after surgery
2017	Schøler *et al*.^[71]^	CRC	Patient-specific mutations for ctDNA	Postoperative detection of residual disease; prognosis for high risk of relapse; early detection of relapse and response to treatment
2017	Chaudhuri *et al*.^[72]^	NSCLC stage I, II, III	Gene panel with CAPPseq ctDNA	Lead time 5.2 months before clinical recurrence
2018	Mehra *et al*.^[73]^	mCRPC, Prostate cancer	cfDNA concentration	Independent prognostic factor for rPFS and OS in first- and second-line chemotherapy
2018	Annala *et al*.^[74]^	mPRPC	ctDNA	Disruption of TP53, BRCA2 or ATM results in worse outcomes on novel AR targeting
2019	Coombes *et al*.^[75]^	Early-stage breast cancer	Signatera assay, Personalized, 16-plex assays for patients-specific mutations in ctDNA	Lead time 8.9 months (up to 2 years) ahead of clinical relapse)
2019	Garcia-Murillas *et al*.^[76]^	Early-stage breast cancer	Monitoring of patient-specific mutations (mutation tracking) by dPCR from plasma cfDNA	Major “proof of concept” supporting the idea of clinical utility for prediction of relapse to be tested in larger studies
2020	Moding *et al*.^[77]^	NSCLC	ctDNA, CAPP-Seq	Detection of MRD with the prediction of benefit from consolidation immunotherapy
2021	McDuff *et al*.^[78]^	LARC	ctDNA	MRD measured by pre- and postoperative ctDNA predicts outcome for chemoradiation
2021	Taniguchi *et al*.^[79]^	CRC	ctDNA	Testing platform combining a prospective screening registry with two phase 3 interventional studies
2022	Gale *et al*.^[80]^	Early stage NSCLC	Monitoring of patient-specific mutations (48 amplicons)	MRD, identification of patients for further therapy
2022	Tie *et al*.^[81]^	Stage II colon cancer	Tumor-informed personalized sequencing	ctDNA-guided approach to reduce adjuvant chemotherapy without compromising recurrence risk

CAPP-Seq: Cancer personalized profiling by deep sequencing; dPCR: digital PCR; LARC: locally advanced rectal cancer; lead time, period before clinical or imaging manifestation of relapse; MRD: minimal residual disease; PPS: patient primary-tumor-specific; TRACERx: TRAcking non-small cell lung Cancer Evolution through therapy (Rx); rPFS: radiological progression-free survival; mCRPC: metastatic castration-resistant prostate cancer.

### Breast cancer

Globally, breast cancer is the most frequent cancer and the leading cause of cancer-related death in women, although most patients can be cured at an early stage. After early surgical removal, additional treatments with chemo-, radio, and/or hormone therapy may further prevent a metastatic relapse. However, overtreatment of cancer-free patients also represents a risk of unnecessary side effects. Therefore, patients should benefit from personalized therapies adjusted for as many additional treatments as required and as few as possible. The challenge to identify MRD with ctDNA analysis from blood samples has been addressed in clinical settings.

In a pilot study in 2015, later complemented with a larger cohort in 2019, Garcia-Murillas and colleagues were able to monitor early breast cancer after initial therapy by detection of ctDNA, which was associated with a high risk of relapse in all breast cancer subtypes^[66,75]^. Individual somatic mutations were identified by sequencing of DNA from the primary tumors. The same mutations were later tracked by digital PCR (dPCR) in patient’s blood samples. Interestingly, the lead time was 10.7 months (95%CI, 8.1-19.1 months), i.e., long before the clinical manifestation of relapse.

Metastatic breast cancer is only rarely curable at a symptomatic stage. In a retrospective study and long follow-up in 2015, Olsson and colleagues combined whole-genome sequencing of primary tumors with personalized dPCR from plasma ctDNA for quantification of tumor-specific chromosomal rearrangements^[67]^. With this method, they were able to detect 0.01% of tumor DNA content or one rearranged sequence per 10,000 wild-type sequences. With an average lead time of 11 months (range 0-37 months), they were able to detect metastatic recurrence accurately before its clinical manifestation. In addition, long-term disease-free survivors had undetectable ctDNA. This supported the rationale to evaluate ctDNA in larger studies to monitor early metastasis detection, adjust therapy, and avoid overtreatment.

Mutations in the tumor suppressor protein p53 (TP53) are common among most cancers, although with varying frequencies^[82,83]^. A high prevalence in a subgroup of breast cancers allowed for monitoring those mutations in ctDNA. Using massively parallel sequencing (MPS), Riva and colleagues detected patient-specific mutations in tumor tissue of non-metastatic triple-negative breast cancer (TNBC)^[84]^. They tracked these patient-specific TP53 mutations in 10 mL of plasma from different time points: (1) before neoadjuvant chemotherapy (NCT); (2) after one cycle; (3) before surgery; and (4) after surgery. With dPCR of ctDNA, a sensitivity of 75% at baseline was achieved. ctDNA levels correlated not only with tumor burden but also with tumor proliferation rate (tumor grade or mitotic index). During NCT treatment, ctDNA levels dropped in all patients. After surgery, no MRD was detectable. However, a shorter survival was correlated with a slow decrease of ctDNA during NCT^[84]^. Radovich and colleagues confirmed ctDNA and CTC enumeration above standard clinical parameters as independent factors for the worse outcome, i.e., distant disease-free survival (DDFS), DFS, and OS [Table 4]^[85]^.

**Table 4:** Examples of clinical trials using ctDNA to validate screening tests or to monitor treatment response

**Year **	**Study**	**Tumor**	**Name**	**Outcome measures**
2014^[85]^	NCT02101385^[86]^	TNBC	Randomized controlled trial of genomically directed therapy in patients with TNBC	Comparison of 2-year DFS rate in patients with a genomically directed therapy or standard of care following preoperative chemotherapy
2019	NCT03934866^[87] ^ Observational study (25,000 participants, 55-77 years with smoking history)	Multiple types of cancer (smoking history), lung cancer	SUMMIT - Cancer screening study using GRAIL’s blood test	Validate blood tests for early detection of multiple types of cancers Examine the performance of LDCT screening
2020	NCT04089631^[88]^ Ongoing interventional clinical trial Phase 3 Drug: Capecitabine	Colon cancer stage II	CIRCULATing tumor DNA-based decision for adjuvant stage II Evaluation (CIRCULATE)	*Primary outcome measure:* DFS of ctDNApos patients; chemotherapy *vs.* follow-up *Secondary outcome measures:* OS in ctDNApos patients with adj. therapy *vs*. follow-up (5 yrs) DFS in ctDNAneg patients (3 yrs) OS in ctDNAneg patients; Kaplan-Meier (5 yrs) DFS and OS of ctDNApos *vs*. ctDNAneg (3 and 5 yrs) Site of metastasis (5 yrs); lymph node *vs*. peritoneal/local recurrence *vs*. other ctDNApos *vs*. ctDNAneg
2021	NCT04931732^[89]^ Observational study	Glioma	circTeloDIAG	Sensitivity and specificity for diagnosis and follow-up

ctDNApos: ctDNA positive; ctDNAneg: ctDNA negative; DFS: disease-free survival; GRAIL: company name; LDCT: low-dose computed tomography; OS: overall survival; SUMMIT: name of study, not an acronym; TNBC: triple-negative breast cancer.

### Colorectal cancer

Colorectal cancer is the second and third most common cancer in women and men, respectively, with almost two million new cases each year^[90]^.

In 2016, Tie and colleagues investigated a prospective cohort of patients after resection of stage II colon cancer^[24]^. They used massively parallel sequencing to identify patient-specific mutations from primary tumors and designed personalized Safe-SeqS assays for the identified mutations to quantify ctDNA from plasma. The presence of ctDNA in 7.9% of patients not treated with adjuvant chemotherapy was highly associated with recurrence of the disease within the median follow-up of 27 months (79% of patients). In patients who completed chemotherapy, the detection of ctDNA also correlated with a worse outcome. Therefore, ctDNA is useful to identify MRD and the high risk of recurrence in patients with stage II colon carcinoma.

Ng and colleagues developed a patient primary-tumor specific (PPS) assay to detect ctDNA in plasma samples by tracking individual mutations identified from tumor tissue^[70]^. ctDNA correlated well with clinical treatment outcome: ctDNA was detectable before, but not directly after, surgery. Furthermore, ctDNA was detectable ahead of clinical manifestation of recurrence, indicating its validity to detect metastasis earlier than established methods of imaging and other markers (CEA).

Schøler and colleagues also showed that ctDNA can be useful for monitoring patients with CRC^[71]^. The detection of ctDNA after surgery reflected MRD and identified patients with a very high risk of relapse. Follow-ups of three years allowed early detection of relapse with a lead time of 9.4 months compared to standard medical imaging, as well as monitoring additional treatment responses. In contrast to CEA levels, preoperative ctDNA detection rates correlated well with the stage of disease. 

For example, CIRCULATing tumor DNA-based decision for adjuvant stage II Evaluation (CIRCULATE), as an ongoing multicenter, prospective, randomized, controlled interventional trial (NCT04089631; Table 4)^[88]^, evaluates the adjuvant therapy in patients with colon cancer stage II. The primary outcome of this phase 3 study is to compare the DFS in patients who are positive for ctDNA with vs. without the drug capecitabine. Patient-specific mutations previously identified from panel analysis of FFPE tumor blocks with a lack of microsatellite instability are used to detect ctDNA as inclusion criteria for the study, thus demonstrating the integration of ctDNA in clinical settings. The study started in June 2020 with expected primary completion in June 2023 and completion in June 2026^[88]^.

### Lung cancer

Lung cancer is the leading cause of cancer death (18.4% of total cancer deaths) and, for both sexes combined, the most common cancer (11.6%) with about 2.1 million cases each year^[90]^. In a seminal paper, Abbosh and colleagues used a phylogenetic approach to track the genetic dynamics of a tumor^[69]^. They identified predictors of ctDNA in early-stage NSCLC: non-adenocarcinoma histology, necrosis, high proliferative index, and lymphovascular invasion. Chaudhuri and colleagues used a gene panel of 128 genes for CAPP-Seq to predict recurrence with an average lead time of 5.2 months^[72]^. In 2016, the FDA broadened the approval of the Cobas test (Roche) to be used not only in tissue sections but also as the first liquid biopsy assay for treatment selection to identify specific EGFR mutations in cfDNA of advanced NSCLC patients [Table 4]^[29]^.

The SUMMIT study at the University College London hospitals plans to enroll 25,000 participants 55-77 years of age who have no diagnosis of cancer but a high risk for lung cancer due to a significant smoking history^[87]^. This study is an ongoing, prospective, observational, cohort study with two major aims: (1) to validate the collaborating company’s (GRAIL, LLC) blood test on cell-free nucleic acids (cfNAs) for early detection of multiple types of cancer; and (2) to deliver low-dose CT (LDCT) screening for lung cancer. Participants will receive at least one low-dose chest CT at baseline. Another possible scan after 12 months will be randomized. The study aims to keep most scans below 1 mSv radiation dose (ultra-low dose) and all scans under 2 mSv. The liquid biopsy will evaluate the test performance, including sensitivity, specificity, and tissue of origin of detected cancers. The performance of the LDCT screening to detect lung cancers will be evaluated in comparison to established measures of risk prediction of lung cancers and other incidental findings. The study started in April 2019 with an estimated primary completion date of August 2023 and an estimated completion date of August 2030^[87]^.

### Prostate cancer

Prostate cancer is the second most common non-cutaneous cancer in men worldwide with an estimated 1.3 million new cases each year with more than 350,000 deaths^[90]^. Prostate cancer contributes to 3.8% of all cancer deaths and is diagnosed in 7.1% of all cancer patients. Mehra and colleagues detected the levels of cfDNA in mCRPC patients in phase 3 clinical studies with first- and second-line chemotherapies^[73]^. Baseline cfDNA concentration, which is partially derived from the tumor, was an independent prognostic factor in both first- and second-line chemotherapy settings, and it correlated with known prognostic factors, shorter radiological PFS (rPFS), and OS. Higher levels of cfDNA before chemotherapy were associated with more aggressive tumors. A decrease of cfDNA within the first few weeks after initiation of chemotherapy correlated with a benefit for the patient, thus identifying a treatment response. Other studies found that cfDNA can include 15%-20% of ctDNA, depending on tumor stage and tumor burden, but localized prostate cancers typically remain below the threshold for detection of ctDNA, whereas prostate-specific antigen (PSA), a protein marker that is not uniquely an indicator for cancer, is already at a high-risk level^[91]^. This implies that cfDNA may currently not be feasible to detect and monitor early state or less aggressive tumors. Annala and colleagues^[74]^ investigated cfDNA from mCRPC patients prior to novel AR therapy. Using whole-exome sequencing (WES) with capturing of coding regions of 72 selected genes, they correlated mutations of TP53, BRCA2, or ATM as predictors of worse outcomes on novel AR targeting, thus suggesting liquid biopsy as new guidance in AR-targeted therapy in general practice.

### Brain tumors

Cancers of the brain and nervous system represent only 1.6% of all cancers and contribute to 2.5% of all cancer deaths. The critical location within the brain and the lack of major improvements for one of the most devastating cancers, glioblastoma, for over a century represent major challenges. Recently, Eibl and Schneemann published a review on liquid biopsy of primary brain tumors^[31]^. In contrast to tumors outside of the nervous system, the blood–brain barrier (BBB) may add another challenge for detecting low-level ctDNA in the blood. CSF-although also protected by the BBB-appears to be a much better source due to the normally diminished number of leukocytes as a background source for cfDNA. However, earlier studies were able to confirm specific mutations known from tissue biopsy also in the serum^[10,21,92,93]^, plasma^[94-96]^, or both^[21]^. In 1991, one of the authors described the very first TP53 mutations in primary medulloblastoma tissue biopsies^[82]^. This finding supports a model of histologically indistinguishable from primitive neuroectodermal tumors (PNET)^[97,98]^. Others were unable at that time to detect TP53 mutations in tissue biopsies or in xenografts of human medulloblastoma, except in only one cell line^[99]^; however, this mutation may have been developed as a selective advantage during cell culture. Similar brain tumor models helped to elucidate and confirm several other oncogenic pathways in human brain tumors^[98,100-107]^. Meanwhile, TP53 mutations in medulloblastomas are well established and can be used as a prognostic and diagnostic marker: only recently, in 2016^[34]^ and with an update in 2021^[108]^, the World Health Organization (WHO) introduced four new diagnostic groups of this childhood brain tumor based solely on molecular genetic features. The correlation between different biological behavior and personalized risk assessment may allow preventing harmful radiation when not necessary or useful. The first two groups refer to different oncogenic signaling pathways, namely wingless/Integration-1 (WNT)-activated (Group 1) and sonic hedgehog (SHH)-activated (Group 2). WNT is a portmanteau for the Drosophila gene “wingless” (Wg), detected in mutants lacking wings, and the homologous mouse gene, integration 1 (Int-1), which was found earlier to cause tumors by insertional mutagenesis with a retrovirus; SHH refers to the hedgehog gene (hh) found in Drosophila mutants with spikes, reminiscent of a hedgehog (SHH is a vertebrate homolog and named after a character in a video game, Sonic the Hedgehog). WNT-activated medulloblastoma shows the highest five-year survival and a low prevalence of metastatic diseases. SHH-activated medulloblastoma can be further separated into two different subgroups, TP53-mutant or TP53-wildtype. SHH-activated, TP53-mutant occurs primarily in older children and has a very poor prognosis, whereas SHH-activated, TP53-wildtype, which is most common in adolescents and young children, has a good prognosis. The other two groups are non-WNT/non-SHH, Groups 3 and 4, respectively (also known as Groups C and D). Group 3 shows an increased prevalence of metastatic disease with the poorest five-year survival, whereas Group 4 has an increased prevalence of metastatic disease with a moderate five-year survival. TP53 mutations in SHH medulloblastomas are associated with poor survival and treatment failures^[34]^. Several subgroups have been associated with TP53 and other mutated genes: for WNT-activated, CTNNB1 and APC; for SHH-activated, TP53, PTCH1, SUFU, SMO, MYCN, and GLI2 (methylome); and for non-WNT/non-SHH, MYC, MYCN, PRDM6, and KDM6A (methylome). Since the WHO classification suggests that the diagnosis from molecular profiling of a tissue biopsy is even superior to classical histopathology, at least for brain tumors, it appears reasonable to use ctDNA-based liquid biopsy for monitoring such mutations in brain tumor patients to avoid repeated and risky neurosurgical biopsies^[109]^. Newer studies successfully used panels of genes. For brain tumors, CSF offers another chance to find ctDNA with a higher sensitivity than plasma or serum^[110-114]^. ctDNA from CSF even represents the genomic mutations better than plasma; CSF shows an increased sensitivity for putative actionable mutations and CNA (copy number aberrations; EGFR, PTEN, ESR1, IDH1, ERBB2, and FGFR2)^[115]^. Glioblastoma (glioblastoma multiforme, GBM) represents the most malignant brain tumor. Even after complete surgical removal, the tumor always relapses due to locally infiltrating cells. Chemo- and radiotherapy treatments can help temporarily, but they also trigger the evolution of the tumor to escape all current treatments. Repeated resection and biopsies are generally not indicated. Therefore, a real-time liquid biopsy with ctDNA from CSF offers an alternative at lower risk to monitor the molecular adaptations of the tumor. CSF as a source for ctDNA also provides an additional chance to investigate brain metastases from tumors from outside of the brain better, since they may contribute to the ctDNA in the blood to a lesser extent than metastases from outside of the brain.

## ctDNA TESTS

Several ctDNA tests have been developed over the past few years. The spectrum of applications includes early screening, detection of targetable mutations to predict treatment response, MRD, and monitoring after treatment with early detection of resistance [Table 5]^[132]^. After extraction of DNA from plasma, the obtained cfDNA is typically used with qualitative PCR or sequencing methods. Such tests can be highly personalized and target varying numbers of tumor-associated or actionable mutations; they can use a custom-built approach to profiling tumor tissue first for patient- and tumor-specific mutations and later use dPCR techniques to monitor tumor development and treatment response from repeated ctDNA probes.

**Table 5:** Examples of ctDNA tests for tumor screening, monitoring, and guiding treatment

**Test**	**Method**	**Genes**	**Cancer type**	**Comments**
Bluestar Genomics^[116-118]^	Hydroxymethylome, NGS, AI	Abnormal genomic/epigenomic signature; 5hmC	Pancreatic cancer (breast and lung cancer)	Screening of patients with new-onset of diabetes, BDD
CancerSEEK^[26]^	ctDNA plus 8 protein markers	ctDNA: 61 Amplicons	Ovary, breast, liver, and others	Sensitivity of about 70% for all 8 cancer types (33%-100% variability)
CellMax-LBx^[119]^	NGS	Mutation profile of 73 genes	Solid tumor	Associated with cancer treatment and tumor response
Circulogene^[120]^	NGS	Approximately 3,000 mutations in > 50 genes	A broad range of tumors	CAP, CLIA
Cobas EGFR mutation test v2^[29,49]^ - (Roche)	PCR, actionable EGFR mutations (exons18-21)	cfDNA	Metastatic NSCLC	FDA approved for plasma/liquid biopsy 2016 to identify patients for the first Erlotinib treatment
DETECT-A^[27]^ – Thrive (developed into: MCED - Exact Sciences)^[121]^	Early version of CancerSEEK^[26]^ ctDNA plus protein markers	ctDNA	Lung, ovarian, CRC and others	1 yr prospective study detecting 26 cancers first in blood
FoundationOne Liquid CDx (Roche Foundation Medicine)^[122]^	NGS	> 300 genes	NSCLC Prostate, ovarian, breast cancer	FDA approved companion diagnostic for treatment
Guardant360 CDx^[123,124]^	NGS	74 genes	Any advanced solid tumors	FDA approved (for 55 genes) tumor mutation profiling
Invitae^[125,126]^ (former ArcherDX)	NGS	ctDNA	Most tumor types	Originally for FFPE, also for ctDNA
Oncomine Pan-Cancer Cell-Free Assay^[127,128]^ - ThermoFisher	NGS	cfDNA 52 genes	Various tumors	CE, Europe
RaDaR - Inivata^[80,129]^	NGS	Up to 48 genes	Early stage cancer, NSCL	Predicts early relapse, MRD, BDD
Signatera^[82,130]^ - Natera	NGS	ctDNA	A broad range of solid tumors	Custom-built personalized MRD assay; BDD
Target Selector - Biocept^[131]^	NGS	18 hotspot genes; 10 (breast) and 11 (lung)	Lung and breast cancer	Focus on actionable genes
Therascreen PIK3CA (Qiagen)^[52]^	PCR	11 activating mutations in exons 7, 9, and 20 of PIK3CA gene	Breast cancer	FDA approved, Identification of patients eligible for PIQRAY (alpelisib)

5hmc: 5-hydroxymethylcytosine; AI: artificial intelligence (machine learning); BDD: breakthrough device designation (by U.S. FDA, Federal Drug Administration); CAP: College of American Pathologists; CE: Communauté Européenne; cfDNA: cell-free DNA; CLIA: Clinical Laboratory Improvement Amendments; DETECT-A: detecting cancers earlier through elective mutation-based blood collection and testing; FFPE: formalin-fixed paraffin-embedded; MCED: multi-cancer early detection; PIK3CA: phosphatidylinositol-3-kinase catalytic subunit alpha; RaDaR: residual disease and recurrence.

### Tumor screening

In a large study, the “Detecting cancers Earlier Through Elective mutation-based blood Collection and Testing” (DETECT-A) blood test demonstrated the proof-of-concept to use ctDNA for tumor screening. The DETECT-A test represents an early version of the CancerSEEK test [Table 5] with minor differences, such as using pre-defined thresholds for each DNA and protein biomarker instead of employing artificial intelligence (AI)/machine learning for increasing sensitivity, or for confirming or enhancing specificity^[27]^. With a cohort of about 10,000 women between 65 and 75 years of age with no history of cancer and one year of follow-up, 26 cancer patients were considered with positive blood testing to be “first detected by blood testing” after confirmation by positron emission tomography-computed tomography (PET-CT): nine lung cancers, six ovarian cancers, and two colorectal cancers - most of them localized or regional. Overall, 14 of the 26 cancers had elevated ctDNA levels, 11 had elevated protein marker levels, and 1 had both elevated ctDNA and protein markers. This test was not designed for regulatory approval, but it addressed fundamental issues of feasibility and safety of multi-cancer blood tests. Indeed, the authors confirmed that a minimally invasive ctDNA-based test was able to safely detect several types of cancer in patients who had no previously known cancer. This allowed early treatment with the intent to cure. The test also produced 101 false positives and 46 false negatives, which still may allow its use as complementary to standard screening methods, although it cannot be used as a stand-alone test. With the use of AI and technology evolution, further improvements appear to be possible.

### Tumor monitoring and treatment guidance

Only a few ctDNA tests are FDA approved for guiding treatment choices to identify cancer patients for mutation-specific treatments: Cobas, FoundationOne Liquid CDx, Guardant 360 CDx, and Therascreen PIK3CA [Table 5]. As an example of detecting targetable mutations in cancer patients, the Cobas EGFR mutation test v2 (Roche) aims to detect by PCR from plasma in less than 4 h specific and actionable mutations in the EGFR gene exons 18-21^[49]^. The FDA approved this test in 2016 for use in liquid biopsy to identify patients for first-line EGFR-targeted therapy (e.g., erlotinib, a tyrosine kinase inhibitor (TKI)) of metastatic NSCLC patients. A similar test was approved earlier for FFPE samples from tumor tissue. In contrast to more recently developing ctDNA tests, the first CTC test approved by the FDA, in 2004, was CellSearch (Veridex). That test uses size, density, electrical properties, and immune surface markers (EpCAM+, Cytokeratins+, and CD45-) for selection and has been cleared for breast, colorectal, and prostate cancer to predict outcome^[12]^.

## VISION AND CHALLENGES

As a new milestone in precision medicine, ctDNA-based liquid biopsy has shown its principal utility and safety for an emerging new era of clinical applications. Nevertheless, a few major challenges need to be addressed within the next few years to reach a widespread benefit for cancer patients: the sensitivity of ctDNA diagnostics may increase by technology improvements, thus allowing even earlier detection and, hopefully, easy-to-use multi-cancer screening platforms for at least the most common cancers. A focus will lie on those as well as developing better treatment options including with the intent to cure. Simple tests, such as detecting only actionable mutations, e.g. EGFR exon mutations, may be further developed for not yet used genes, for example, rare and tumor-specific CD44v variant exon combinations^[133]^. Although many studies describe a significant “lead time” to detect a relapse before any clinical methods or radiologic imaging, one should reflect that any of these clinical methods may also improve over time, e.g., a 3 or 7 Tesla (T) MRI should have a better resolution and detection capacity than a 1.5 T MRI, thus pointing to the uncertainty of comparisons over time. Hundreds of recent and ongoing clinical trials already apply ctDNA methods to monitor new treatments in cancer patients^[65,134]^. Large multicenter studies will further show how to best standardize and incorporate the use of ctDNA in clinical guidelines. Other arms of liquid biopsy may also be improved in parallel. Living CTCs should be further characterized by not yet applied technologies, such as functional assays with quartz-crystal microbalance (QCM)^[135]^ or atomic force microscopy (AFM), e.g., to check for tumor-specific cell adhesion and pharmacology reactions at the single-molecule level, both applied and pioneered by one of the authors^[136-144]^. Sharing pharmacological and migratory data on CTCs, and making them findable, accessible, interoperable, and reusable (FAIR), will allow meta-analysis, data integration, and data mining to accelerate such new approaches in liquid biopsy^[145]^. Some of these experimental technologies may be tested in established models first to allow better reproducibility^[146,147]^. CellSearch with counting CTCs became clinically validated as the first CTC marker for worse outcomes in early breast cancer^[148]^. It appears reasonable to combine such CTC tests with ctDNA methods to improve liquid biopsy, perhaps similarly to how CancerSEEK successfully combined ctDNA mutations with protein markers.

## CONCLUSION

Depending on tumor type and treatment options, ctDNA has been shown to be a valid and independent biomarker for confirming and serial monitoring of cancer. With its very high specificity, ctDNA can be used to predict the early recurrence of a wide variety of cancers after initial therapy. This enables guidance to earlier and better treatment options, including the intent to cure, but also to prevent patients from unnecessary treatments, when there is no indication for MRD or early detection of resistance development. Numerous larger and ongoing clinical studies with ctDNA as a marker for monitoring MRD or treatment response will increase our knowledge on how to apply the best strategies.

Unfortunately, sensitivity often appears to be too low to use ctDNA for general tumor screenings, and not all patients can be monitored reliably. Further improvement in technology is needed to increase sensitivity and standardization. Although ctDNA appears to be much closer to widespread routine use than the other arms of liquid biopsy (CTCs, EVs, and TEPs), perhaps, a combination of ctDNA data with functional studies on living CTCs can soon close the gap for a faster shift to the new paradigm of liquid biopsy in personalized medicine for the benefit of most cancer patients at any stage of diseases. ctDNA as a valid biomarker is ready to enter clinical routine.

## DECLARATIONS

### Copyright

© The Author(s) 2022.
